# Beyond pillbox: a national cross-sectional study on the attitudes, practices, and knowledge of community pharmacists regarding complementary and alternative medicine

**DOI:** 10.1186/s12906-024-04391-8

**Published:** 2024-02-28

**Authors:** Farah Naja, Nada Abbas, Sharfa Khaleel, Falak Zeb, Tareq A. H. Osaili, Reyad Shaker Obaid, MoezAlIslam Faris, Hadia Radwan, Leila Cheikh Ismail, Haydar Hassan, Mona Hashim, Hamzah AlZubaidi

**Affiliations:** 1 Department of Clinical Nutrition and Dietetics, College of Health Sciences, University of Sharjah, P.O. Box 27272, Sharjah, United Arab Emirates; 2 Research Institute of Medical and Health Sciences, University of Sharjah, P.O. Box 27272, Sharjah, United Arab Emirates; 3 Department of Nutrition and Food Sciences, American University of Beirut, P.O.Box 110236, Beirut, Lebanon; 4 Department of Nutrition and Food Technology, Faculty of Agriculture, Jordan University of Science and Technology, P.O. Box 3030, Irbid, 22110 Jordan; 5 Department of Pharmacy Practice and Pharmacotherapeutics, University of Sharjah, P.O. Box 27272, Sharjah, United Arab Emirates; 6 School of Medicine, Deakin Rural Health, Deakin University Faculty of Health, Warrnambool, VIC 3216 Australia

**Keywords:** Complementary medicine, Alternative Medicine, Community pharmacists, United Arab Emirates, Knowledge, Attitude, Practice

## Abstract

**Background:**

While community pharmacists are uniquely positioned to promote the safe and effective use of complementary and alternative medicine, their potential role remains largely untapped. The objective of this study was to assess the knowledge, attitudes, and practices related to complementary and alternative medicine products among community pharmacists in the United Arab Emirates and explore the correlates of knowledge in the study sample.

**Methods:**

Using a stratified random sampling frame, a national survey of community pharmacists was conducted in the United Arab Emirates. In a face-to-face interaction, participants completed a multi-component questionnaire consisting of four sections: sociodemographic and general characteristics; knowledge of complementary and alternative medicine products and usage; attitudes towards complementary and alternative medicine and practices related to complementary and alternative medicine. Based on participants’ answers, scores were calculated with higher scores indicating more positive knowledge, attitudes, and practices.

**Results:**

373 community pharmacists participated in the study (response rate: 83%). For the knowledge questions, more than 50% of community pharmacists correctly answered the functions of complementary and alternative medicine, however lower percentages were noted for the side effects and drug interactions questions. Most community pharmacists had positive attitudes towards complementary and alternative medicine, except for particular aspects such as efficacy, where 40% agreed that complementary and alternative medicine is only effective in treating minor complaints. As for practices, while more than 70% of participants counseled patients on complementary and alternative medicine use, only 47% reported the toxic effects when encountered. Multiple linear regressions showed that community pharmacists working in independent pharmacies, those with fewer years of experience, and those who did not receive complementary and alternative medicine education during their academic degree had lower knowledge scores (*p* < 0.05).

**Conclusions:**

The findings of this study showed that community pharmacists in the United Arab Emirates have good knowledge of complementary and alternative medicine functions and generally positive attitudes and practices, with few gaps identified in each. Together, these findings provide critical evidence for the development of targeted interventions to promote the role of community pharmacists towards safe and effective complementary and alternative medicine use in the country.

## Background

Complementary and Alternative Medicine (CAM) comprises a broad set of healthcare approaches and substances that are not integrated fully into dominant healthcare as conventional medicine [1]. Such approaches and substances are defined as ‘alternative’ when used in place of conventional medicine and ‘complementary’ when used together with conventional medicine. According to the National Center for Complementary and Alternative Medicine (NCCAM), CAM has five main categories: mind body therapies, manipulative and body-based methods, energy therapies, whole medical systems as well as biologically based-therapies. The latter includes herbal extracts, animal-derived extracts, vitamins, minerals, fatty acids, amino acids, proteins, prebiotics and probiotics, whole diets, and functional foods [2]. For the purpose of this study CAM refers to the biologically based therapies, the most commonly used for of CAM.

Since the turn of the century, the interest in CAM has been steadily increasing, as the world faces the menacing health challenges of non-communicable diseases, infectious diseases, mental health disorders, age-related conditions and unequal access to healthcare [3]. A recent systematic review of national studies addressing the prevalence of CAM use by the general population reported a prevalence ranging between 24% and 71% [4]. Higher use rates are reported in Africa and South Asia (80% and 97%) [5, 6]. Many reasons are postulated to have led to this revival in CAM utilization including dissatisfaction with conventional medicine, a feeling of patient empowerment and personal responsibility through active participation in one’s own health and wellness, and a belief that CAM therapies have fewer side effects and are cost-effective compared to conventional medicine [7]. This prevalent use of CAM products is challenged by issues regarding their safety. Potential toxicities, adverse reactions, or interactions with conventional treatments are commonly reported alongside the use of many CAM products [8]. These risks are compounded by the fact that many CAM users do not disclose their practices to their healthcare providers for reasons of mistrust or fear of disapproval [9, 10].

Among healthcare professionals, pharmacists are uniquely positioned to promote safe and effective, use of CAM [11]. Pharmacists are trusted and accessible healthcare professionals who, in many instances, are labelled as “gatekeepers” of medications. The pharmacists’ involvement at the point of sale is therefore critical to help consumers make informed decisions about CAM products [12]. Professional pharmacy organizations such as such as the American Society of Health-System Pharmacists (ASHP), the Canadian Society of Hospital Pharmacists and the American College of Clinical Pharmacy (ACCP) have recognized and endorsed the central role of pharmacists in CAM use [12]. For instance, the ACCP issued a position statement stating that ‘the pharmacist’s involvement in herbal products is an extension of their roles in pharmaceutical care, clinical pharmacy practices and collaborative healthcare teams’ [13–15]. These recommendations have led many countries to integrate CAM within the professional competency frameworks of their pharmacy education systems. For instance, in Ireland, the National Competency Framework for Pharmacists Working In Cancer Care included distinct competencies for CAM practice at the generalist, specialist and advanced specialist pharmacy levels [16]. A scoping review of the competency-based pharmacy education in the Eastern Mediterranean Region showed that studies from Saudi Arabi, Kuwait and Pakistan highlighted the need to incorporate CAM courses in the pharmacy education system [17]. However, the gap remains between the professional recommendations and current pharmacy practice standards regarding CAM products. Lack of education and training in the field, limited regulations surrounding CAM products, and inadequate availability of resources in the pharmacy are among the challenges faced by pharmacists [18, 19].

Within the Middle East and North Africa (MENA) region, the United Arab Emirates (UAE) houses the fastest-growing market for CAM [20]. Varying rates of CAM use were reported in the region, such as 30% among adults in Lebanon [21], 40% among women trying to lose weight in Jordan [22], reaching as high as 72% in the general population of Babol, Iran [23] and 75% among various patient populations in Saudi Arabia [3]. Studies on rates of CAM use among the general population in the UAE reported a range between 40% and 76% [24, 25]. The growing popularity of CAM in the country has prompted the government to set regulatory frameworks for CAM products, under two different categories: either as over-the-counter (OTC) medications, which are dispensed by a pharmacist without needing a physician’s prescription, or Prescription Only Medicines (POM) [26]. For both categories, the sale of the CAM products takes place mostly at the community pharmacy. A pilot study on the role of pharmacists in CAM use conducted in Abu Dhabi, the capital of the UAE and one of its seven Emirates, revealed a heavy involvement of pharmacists in this field, whereby 85% of surveyed pharmacists indicating always, often or sometimes dispensing CAM products and/or counselling patients on the safe use of the CAM products. This pilot study highlighted the need for a national investigation on the role of pharmacists in CAM use to promote their roles in ensuring a safe and effective use of CAM in the country [26].

The primary objective of this study was to assess the knowledge, attitudes and practices related to CAM products among a national sample of community pharmacists in the UAE. The secondary objectives of the study were to investigate the associations of perceived versus actual knowledge of CAM and to explore the correlates of knowledge among pharmacists in the UAE. The findings of this study will provide the needed evidence for formulating policies and recommendations to support the role of pharmacists in ensuring safe and effective use of CAM.

## Methods

This is a cross-sectional national study aiming to explore the knowledge, attitudes, and practices of community pharmacists (CPs) regarding CAM use in the UAE. The study was conducted between October 2022 and April 2023. For the selection of CPs, a stratified random sampling frame was used, with the seven emirates of the UAE constituting the different strata (Abu Dhabi, Sharjah, Dubai, Ras Al Khaimah, Ajman, Fujairah and Umm Al Quwain). Within each emirate, community pharmacies were randomly chosen and the pharmacist available at the selected pharmacy was invited to participate. In the case where more than one pharmacist was available and willing to participate at the pharmacy, only one pharmacist was invited to take part in the study. This pharmacist was selected randomly using the Kish method [27]. A probability proportional to size approach was used to determine the number of CPs from the various strata (emirates) whereby the number of CPs recruited in each emirate was proportional to the number of CPs in this emirate compared to the total number of CPs in the country. The study was reviewed and approved by the Research Ethics Committee of the University of Sharjah (REC-23-02-16-01-F). A signed consent form was obtained from the CPs before enrolling in the study.

Sample size calculations showed that a minimum number of 302 CPs is needed in order to estimate an outcome prevalence of 85% with a 5% margin of error and 95% confidence interval [28]. The prevalence of 85% used in the sample size calculations was based on the results of a pilot study conducted in the Emirate of Abu Dhabi and which found that 85% of surveyed pharmacists indicated dispensing CAM products and/or counselling patients on the safe use of the CAM [26]. To be eligible to participate in the study, the pharmacist had to be conversant in English and possess a valid license to practice pharmacy in the UAE. CPs practicing in either chain or independent pharmacies were included.

Trained field workers conducted data collection across the country. At the pharmacy, the data collectors approached the pharmacist and explained the study objectives and protocol. Interested pharmacists were invited to read and sign the consent form. In face-to-face interactions, the CPs completed a multicomponent questionnaire using a paper and a pen. Prior to data collection, interactive sessions were delivered to train the field workers on the various sections of the questionnaire and the best practices of data collection. During these sessions, hands-on demonstrations and role-play interviews were conducted with an emphasis on data integrity and ethical considerations. Feedback oops for continuous data monitoring were established to ensure data quality.

Data for this study was collected using a multi-section questionnaire addressing the knowledge, attitudes and practices of CPs regarding CAM. A panel of experts, including a pharmacist, a CAM researcher, and a nutritionist developed the questionnaire used in the study, following a four-step approach: question generation, question design and structure, validation process and revision. The question generation relied on an extensive review and analysis of relevant literature by searching key databases (PubMed, Web of Science and Google Scholar) using relevant broad search items such as CPs, knowledge, practices, attitudes, complementary and alternative medicine, nutritional supplements and herbal medicine. In the design step, the questions were crafted using clear and concise language, minimizing ambiguity, and avoiding leading and biased questions. The use of scaling and response options was carried out based on the nature of the data to be collected and ensuring that the range of possible answers was covered. The validation process addressed both the face and the content validity of the questionnaire, whereby the panel of experts ensured that the questions adequately covered the study objectives. The revision step encompassed an internal review by the panel, identifying and addressing any issues related to question wording, clarity, and relevance. In addition, the questionnaire was piloted on 10 randomly selected CPs to test ease of comprehension, clarity, cultural appropriateness and the time needed for completion. As a result, a few questions were revised. For instance, a few pharmacists were not familiar with ‘Joshanda’, hence it was replaced with Mulethi/liquorice. The data obtained from the pilot study was not included in the analysis. The time needed to complete the questionnaire ranged between 15 and 20 min.

The questionnaire consisted of four main sections: The first section was related to the socio-demographic and general characteristics of the CPs such as age, gender, marital status, nationality, highest education qualification, graduation country, pharmacy location, pharmacy setting, employment status, work experience, and education or training regarding CAM. In addition, within this section, questions related to CAM education and primary sources of information about CAM were included. The second section evaluated the perceived and actual knowledge of CPs related to CAM products and usage. Pharmacists were asked to evaluate their knowledge (perceived knowledge) about diet and herbal products, as have minimal knowledge, understand basic principles, or pursue further knowledge. The actual assessment of knowledge included 17 statements related to various CAM therapies and addressed both the functions and the side effects/toxicity/interactions of these therapies. Pharmacists were given the choice to answer these statements as correct, incorrect or I don’t know. A total knowledge score was computed using the CPs answers to the 17 statements, whereby the right answer was assigned 1 point, while the false and I don’t know answers were assigned 0 points. A total knowledge score was computed, with a possible range from 0 to 17, with higher scores indicating better knowledge. The attitudes of CPs regarding CAM were evaluated using 19 questions which addressed the pharmacists’ attitudes regarding their role within the field of CAM, CAM education in pharmacy schools, regulation of CAM market and products, and efficacy and safety of CAM. Participants rated each attitude statement on a 5-point Likert scale, ranging from 1 = strongly disagree, 2 = disagree, 3 = neutral, 4 = agree or 5 = strongly agree. To calculate a score for attitudes, participants were given 1 to 5 points for each statement corresponding to their choices with a higher number of points indicating a more positive attitude. For statements with negative attitudes, a reverse scoring was used. The total score was obtained by summing the points obtained for each of the 19 statements, hence ranging from 19 to 95. The last section of the questionnaire explored the CPs practices related to CAM products. It consisted of nine statements describing various practices such as selling CAM products, informing patients about products’ side effects, asking about medical history and drug interactions, reporting toxic and undesirable effects, in addition to various counselling techniques when dispensing CAM. The pharmacist answered these practices statement with frequencies ranging from 1 = always, 2 = often, 3 = sometimes, 4 = rarely and 5 = never. A similar approach to that of the attitudes score was used to calculate that of practices. As such the practice score ranged from 9 to 45 with higher scores indicating positive practices.

Data were analyzed using STATA version 13. Descriptive statistics were presented as mean and standard deviation (SD) for continuous variables, and frequency and percentage were used for categorical variables. Determinants of higher knowledge scores were examined through simple and multiple linear regression analyses. In the multiple regression analysis, variables with a *p*-value < 0.2 in the simple regression analysis, along with age and gender, were included. The table describing the regression analyses includes crude coefficients, *p*-values, and 95% confidence intervals (CI), as well as adjusted coefficients, *p*-values, and 95% CI. Additionally, the relationship between the knowledge score and perceived knowledge level was assessed using Spearman’s correlation. A Pearson correlation was also used to test the link between knowledge, attitude, and practice scores.

## Results

Out of 450 pharmacists approached, 373 agreed to participate (response rate: 83%). The main reasons for refusing to participate were lack of time and interest in the study objectives. Table 1 presents the sociodemographic and general characteristics of the study population. One in two participants was in the age range of 20–30 years (52.5%) and was a female (56.8%). Most participants were married (64.1%), of non-Arab nationalities (66.5%), and had a bachelor’s degree (76.9%). Graduation countries varied, with 59.2% of participants graduating from South Asian countries, 35.4% from MENA countries, and 5.4% from East Asian and European countries. The study encompassed both independent pharmacies (26.5%) and chain pharmacies (73.5%), with a significant number of pharmacies having three or more pharmacists (57.9%). The average work experience was 6.5 ± 5.0 years. CAM education was integrated into the undergraduate pharmacy curriculum for 77.2% of participants, while 34.3% had received post-graduate education/training on CAM products. The primary sources of knowledge regarding CAM modalities were divided between education/training/work experience (82.04%) and social media/family and friends (17.96%). (Table 1)

**Table 1:** Sociodemographic and general characteristics of the study sample (*n* = 373)

		*n*	%
Age (years)	20–30	196	52.5%
	31–40	149	39.9%
	41–50	23	6.2%
	> 50	5	1.3%
Gender	Male	161	43.2%
	Female	212	56.8%
Marital status	Not married	134	36%
	Married	239	64.1%
Nationality	Arab (including GCC and non-GCC countries)	125	33.5%
	Non-Arab	248	66.5%
Qualification	Bachelors	287	76.9%
	Masters / PhD	52	14%
	PharmD	34	9.1%
Graduation country	MENA countries	132	35.4%
	South Asian countries^*^	221	59.2%
	East Asian and European countries^**^	20	5.4%
Pharmacy location	Abu Dhabi	94	25.2%
	Dubai	124	33.2%
	Sharjah	85	22.8%
	Other emirates (UAQ, RAK, Ajman, and Fujairah)	135	36.3%
Pharmacy type	Independent pharmacy	99	26.5%
	Chain pharmacy	274	73.5%
Current employment	Pharmacist	342	91.7%
	Pharmacist technician / Trainee	31	8.3%
Work experience in years (Mean SD)		6.45	5.04
Number of pharmacists in the pharmacy	1 pharmacist	19	5.1%
	2 pharmacists	138	37%
	≥ 3 pharmacists	216	57.9%
Education on CAM included in undergraduate pharmacy curriculum	No	85	22.8%
	Yes	288	77.2%
Received post-graduate education/training on CAM products	No	245	65.7%
	Yes	128	34.3%
Perceived usefulness of education/training on CAM products	Not Useful	6	1.6%
	Neutral	51	13.7%
	Very Useful	119	31.9%
	No answer	197	52.8%
Willingness to attend trainings/ workshops on CAM products	No	64	17.2%
	Yes	309	82.8%
Primary source of knowledge of CAM modalities (Diet and herbs therapy)	Social Media / Family & friends	67	17.96%
	Education / Training / Work Experience	306	82.04%

^*^Pakistan, India, Bangladesh

^**^Philippines, France and Ukraine

**Fig. 1:**
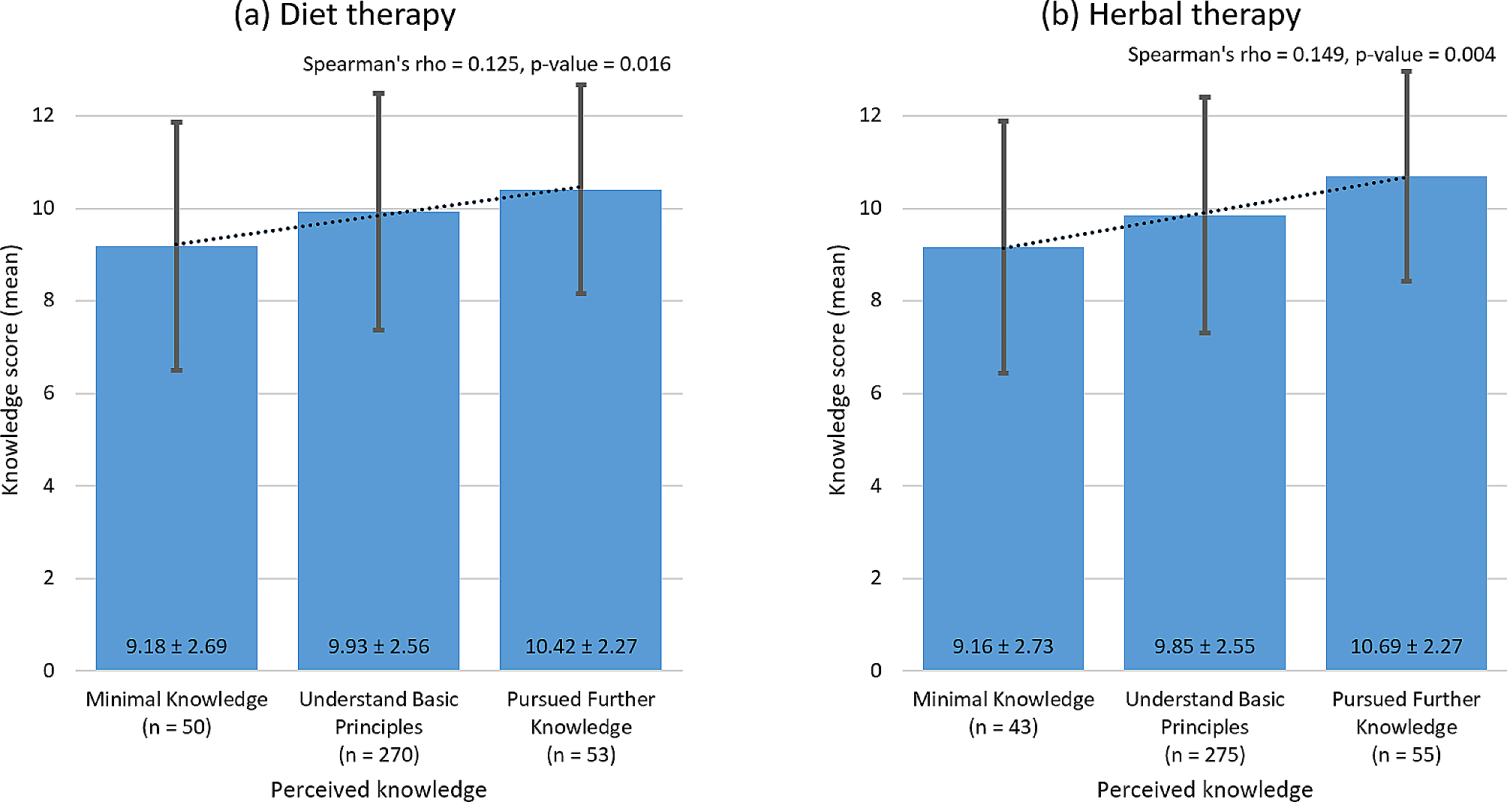
Association of knowledge scores with perceived knowledge regarding **(a)** diet therapy and **(b)** herbal therapy. Greater perceived knowledge in both diet therapy and herbal therapy is linked to higher knowledge scores (Spearman’s rho = 0.125, *p*-value = 0.016 for diet therapy; Spearman’s rho = 0.149, *p*-value = 0.004 for herbal therapy)

Figure 1 depicts the association between knowledge scores and pharmacists’ perceived knowledge levels of diet therapy and herbal therapy. Participants who pursued further knowledge achieved the highest mean knowledge scores for diet and herbal therapy, followed by those who understood basic principles and then those with minimal knowledge. This positive correlation between knowledge scores and perceived knowledge levels in both diet therapy and herbal therapy was statistically significant, with a Spearman’s rho value of 0.125 (*p*-value = 0.016) for diet therapy and a Spearman’s rho value of 0.149 (*p*-value = 0.004) for herbal therapy. (Fig. 1)

**Fig. 2:**
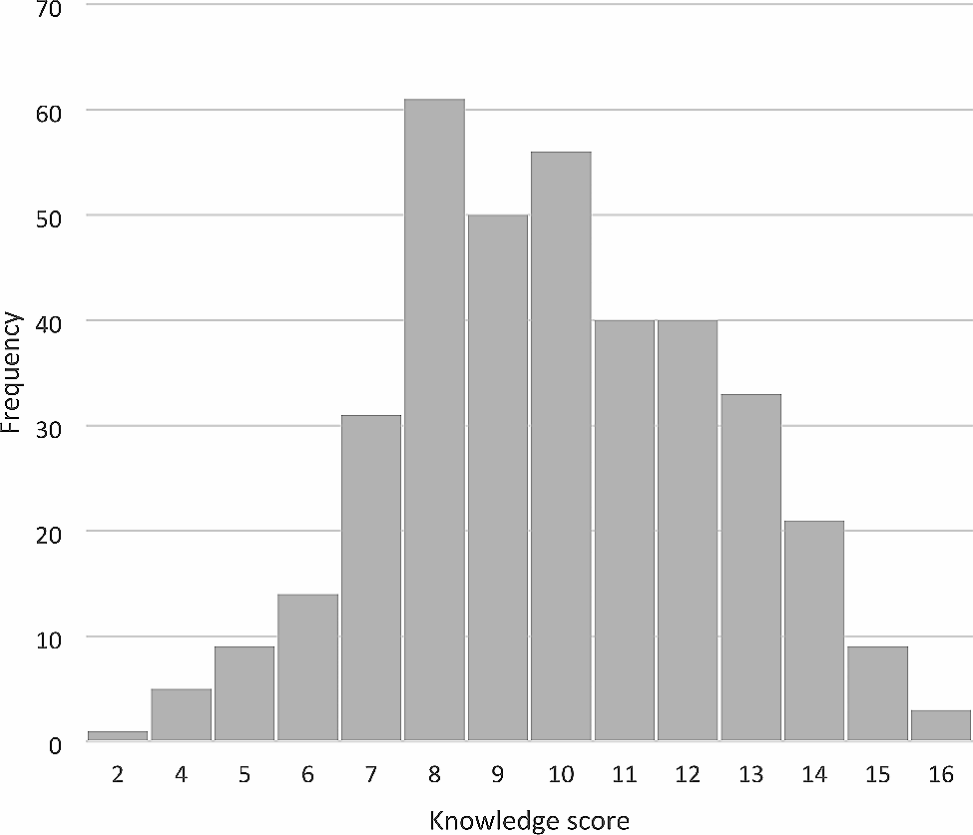
Knowledge score distribution among study participants (*n* = 373)

The mean knowledge score among the study sample was 9.9 ± 2.55. Figure 2 provides a visual representation of the distribution of knowledge scores among the study participants. The distribution appears to be relatively normal, with a slight left skew.

Table 2 presents the knowledge score items in the study sample. The table is divided into two sections: Functions and Side Effects. The majority of participants correctly identified the functions of Omega-3 (96.0%), Echinacea (86.1%), Garlic (86.1%), Ginkgo biloba (77.7%), and Mulethi/Liquorice (77.2%). However, a lower proportion of participants correctly identified the functions of Ginseng (48.0%), Vitamin B (55.0%), and Valerian (57.1%). Overall, participants demonstrated greater knowledge of CAM functions compared to CAM side effects. More than half of the participants recognized that herbal medicine is not without side effects (56.0%). Additionally, most participants were aware of the relationship between vitamin C and iron (77.7%) and the interaction between Omega-3 and clopidogrel (67.0%). However, a lower proportion of participants identified the risks associated with long-term use of Liquorice (53.1%), the potential drug-herb interactions involving Mulethi/Liquorice (45.0%), the interaction between Gingko and Coumadin (30.6%), the safety of St. John’s wort in patients taking cyclosporine (24.4%), and the possibility of insomnia as a side effect of Ginseng (22.3%). (Table 2).

**Table 2:** Description of knowledge score items in the study sample (*n* = 373)

	Correct answer	Correct		False / I do not know	
		*n*	%	*n*	%
***Functions***					
Echinacea is effective against cold and flu symptoms	(Correct)	321	86.1%	52	13.9%
Mulethi/ Liquorice is a commonly used herb for mild to moderate sore throat and cough	(Correct)	288	77.2%	85	22.8%
Garlic can lower blood lipid level	(Correct)	321	86.1%	52	13.9%
Ginseng can be used safely in people with high blood pressure	(Incorrect)	179	48.0%	194	52.0%
Ginkgo biloba is commonly used in people with Alzheimer’s disease	(Correct)	290	77.7%	83	22.3%
Omega-3 is beneficial for patients suffering from cardiovascular disorders	(Correct)	358	96.0%	15	4.0%
Vitamin B complex may delay wound healing	(Incorrect)	205	55.0%	168	45.0%
Valerian should be used cautiously in patients using benzodiazepines	(Correct)	213	57.1%	160	42.9%
***Side Effects***					
Herbal medicine is natural and therefore is safe, without side effects	(Incorrect)	209	56.0%	164	44.0%
Mulethi/ Liquorice may cause drug-herb interactions	(Correct)	168	45.0%	205	55.0%
Long-term use of liquorice is not recommended	(Correct)	198	53.1%	175	46.9%
Omega-3 can be given safely to patients taking clopidogrel	(Correct)	250	67.0%	123	33.0%
Vitamin C when taken with Iron (Ferrous salt) increases its absorption	(Correct)	290	77.7%	83	22.3%
Insomnia is a possible side effect when taking Ginseng	(Incorrect)	83	22.3%	290	77.7%
Gingko increases risk of bleeding in patients taking Coumadin	(Correct)	114	30.6%	259	69.4%
Chronic zinc supplementation is a possible risk factor for anemia	(Correct)	114	30.6%	259	69.4%
St John’s wort is effective and safe for use in patients taking cyclosporine	(Incorrect)	91	24.4%	282	75.6%

Table 3 provides a comprehensive description of the attitudes towards CAM modalities among the study sample. The table displays the responses across different categories ranging from “Strongly Disagree” to “Strongly Agree”. A considerable proportion of participants (72.2%) perceived the use of CAM products to be common among the UAE community. Moreover, there is a consensus among the participants that continuing education related to CAM products should be mandatory for pharmacists, with a majority (82.6%) expressing agreement or strong agreement. The majority also agreed on restricting CAM use to medically qualified personnel (82.6%) and on the importance of consulting a health professional (80.1%), and most disagreed with the statement that herbal medicine is unsafe and ineffective (77.8%). However, there were notable areas where attitudes towards CAM were less positive. For instance, a significant percentage of participants (41%) expressed the belief that CAM has a low status within medicine. Similarly, a substantial proportion of participants (40%) agreed that CAM is only effective in treating minor complaints. Additionally, participants’ responses indicate that there is a perception that practitioners of CAM earn less money compared to other doctors (36%). (Table 3).

**Table 3:** Description of attitudes towards CAM in the study sample (*n* = 373)

	Strongly disagree		Disagree		Neutral		Agree		Strongly agree	
	*n*	%	*n*	%	*n*	%	*n*	%	*n*	%
CAM product use is common amongst the UAE community	9	2.4%	15	4.0%	80	21.4%	177	47.5%	92	24.7%
Continuing education related to CAM products should be mandatory for pharmacists	16	4.3%	8	2.1%	41	11.0%	188	50.4%	120	32.2%
CAM products available in the market are well standardized	16	4.3%	33	8.8%	104	27.9%	167	44.8%	53	14.2%
CAM products available in the market are of good quality	8	2.1%	25	6.7%	104	27.9%	182	48.8%	54	14.5%
All practitioners of CAM should be medically qualified	11	2.9%	8	2.1%	46	12.3%	178	47.7%	130	34.9%
CAM should be taught in pharmaceutical school	70	18.8%	209	56.0%	70	18.8%	16	4.3%	8	2.1%
Increasing number of patients claim CAM is effective at curing their illness	7	1.9%	31	8.3%	111	29.8%	187	50.1%	37	9.9%
CAM is more cost-effective than modern medicine	11	2.9%	104	27.9%	134	35.9%	102	27.3%	22	5.9%
The reason for the success of CAM is mainly due to treating the person as a whole being	6	1.6%	29	7.8%	152	40.8%	154	41.3%	32	8.6%
CAM may have positive effect on general health outcomes	8	2.1%	20	5.4%	73	19.6%	231	61.9%	41	11.0%
Self-care and interest in our own health is one reason that people are drawn to CAM	7	1.9%	21	5.6%	94	25.2%	215	57.6%	36	9.7%
Providing information about herbal medicine is part of a pharmacist’s professional responsibility.	56	15.0%	191	51.2%	82	22.0%	31	8.3%	13	3.5%
It is important to consult a health professional before using CAM	8	2.1%	13	3.5%	53	14.2%	200	53.6%	99	26.5%
On average, practitioners of CAM make less money than other doctorsˠ	14	3.8%	55	14.7%	169	45.3%	103	27.6%	32	8.6%
CAM has a low status within medicineˠ	6	1.6%	68	18.2%	146	39.1%	137	36.7%	16	4.3%
CAM is only effective in treating minor complaintsˠ	12	3.2%	102	27.3%	109	29.2%	137	36.7%	13	3.5%
CAM is fairly unscientificˠ	33	8.8%	161	43.2%	104	27.9%	66	17.7%	9	2.4%
Practitioners of CAM listen to their patients more than other health care professionalsˠ	14	3.8%	62	16.6%	163	43.7%	104	27.9%	30	8.0%
Herbal medicine is unsafe and ineffectiveˠ	76	20.4%	214	57.4%	47	12.6%	30	8.0%	6	1.6%

ˠ used to indicate items where a “Strongly Agree” response is indicative of a negative attitude. For all other items, a “Strongly Agree” response is indicative of a positive attitude towards CAM.

Table 4 provides an overview of the practices related to CAM modalities among the study sample. The table presents participants’ responses ranging from “Always” to “Never” for various practices. The findings indicate that a significant proportion of participants always or often advise on supplement consumption (88.2%), ask about consumer’s medical history when recommending CAM products (74.3%), and check for interactions with prescription drugs (69.7%), inform consumers about the potential risks associated with the use of CAM products (72.4%), inform consumers about the possible adverse effects of dietary supplements (70.8%), sell CAM products in their pharmacies (70.5%) and receive inquiries from patients about CAM product use (65.4%). However, over half of the participants (53.4%) rarely or never report toxic effects from CAM product use. Moreover, a significant proportion of participants reported rarely or never receiving referrals from CAM practitioners (27.4%). (Table 4).

**Table 4:** Description of practices related to CAM products in the study sample (*n* = 373)

	Always		Often		Sometimes		Rarely		Never	
	*n*	%	n*n*	%	*n*	%	*n*	%	*n*	%
Do you sell CAM products in your pharmacy?	148	39.7%	115	30.8%	92	24.7%	11	2.9%	7	1.9%
Do you get inquiries from patients regarding the use of CAM products?	132	35.4%	112	30.0%	100	26.8%	19	5.1%	10	2.7%
Do you inform consumers about the possible adverse effects of dietary supplements?	166	44.5%	98	26.3%	78	20.9%	23	6.2%	8	2.1%
Do you advise consumers on how the supplements are to be consumed?	264	70.8%	65	17.4%	33	8.8%	6	1.6%	5	1.3%
Do you ask consumer’s medical history when recommending CAM products?	201	53.9%	76	20.4%	68	18.2%	16	4.3%	12	3.2%
Do you check whether particular supplement taken by consumer interacts with his/her prescription drugs?	191	51.2%	69	18.5%	80	21.4%	21	5.6%	12	3.2%
Do you inform consumers about the potential risks associated with the use of CAM products (e.g. side effects, drug-herb interactions)?	182	48.8%	88	23.6%	69	18.5%	26	7.0%	8	2.1%
Do you report any toxic or undesirable effects that occur with patients using CAM products?	74	19.8%	41	11.0%	59	15.8%	95	25.5%	104	27.9%
Do you get referrals from CAM practitioners to your pharmacy?	42	11.3%	60	16.1%	108	29.0%	85	22.8%	78	20.9%

Examining the associations of knowledge, attitudes and practices in the study population, the results indicated that only knowledge and attitudes were related (*r* = 0.108, *p* = 0.036); while none was associated with practices. (Data not shown)

Table 5 presents the results of the simple and multiple linear regression analyses examining the association between different characteristics of study participants and their knowledge scores regarding CAM. In the adjusted model, participants aged 41–50 had significantly lower knowledge scores compared to those aged 20–30 (*p* = 0.034). Graduates from South Asian countries had lower knowledge scores compared to graduates from MENA countries (adjusted coefficient = -0.66, *p*-value = 0.028). Participants working in Dubai, Sharjah, and other emirates exhibited a significant positive association with knowledge scores compared to those in Abu Dhabi (*p*-values = 0.009, 0.021, and 0.008, respectively). Working in chain pharmacies was associated with an average knowledge score that was 0.88 points higher compared to independent pharmacies (adjusted *p*-value = 0.004), and each year of work experience was linked to a 0.13-unit increase in knowledge score (adjusted *p*-value = 0.001). Education on CAM within the undergraduate pharmacy curriculum demonstrated a positive association with knowledge scores (adjusted coefficient = 0.68, *p*-value = 0.026). Likewise, having education, training, or work experience as the primary source of CAM knowledge was associated with higher knowledge scores compared to relying on social media and/or friends (adjusted coefficient = 0.79, *p*-value = 0.021). Master’s or PhD degrees showed borderline non-significant positive associations, while PharmD qualification did not exhibit a significant association with knowledge scores.

**Table 5:** Associations of study participants’ characteristics with knowledge, as derived from simple and multiple regressions (*n* = 373)

		Crude			Adjusted		
		Β coef.	*p*-value	95% CI	Β coef.	*p*-value	95% CI
Age	20–30 y						
	31–40 y	**0.62**	**0.026**	**(0.07; 1.16)**	0.13	0.688	(-0.52; 0.78)
	41–50 y	-0.44	0.436	(-1.53; 0.66)	**-1.54**	**0.034**	**(-2.95; -0.12)**
	> 50 y	1.95	0.091	(-0.31; 4.2)	0.14	0.913	(-2.32; 2.6)
Gender	Male						
	Female	0.19	0.472	(-0.33; 0.72)	0.21	0.455	(-0.34; 0.77)
Marital status	Not married						
	Married	0.03	0.921	(-0.52; 0.57)			
Nationality	Arab (including GCC and non-GCC countries)						
	Non-Arab	-0.09	0.74	(-0.64; 0.46)			
Qualification	Bachelors						
	Masters / PhD	0.70	0.068	(-0.05; 1.46)	0.63	0.092	(-0.1; 1.36)
	PharmD	-0.40	0.393	(-1.3; 0.51)	-0.03	0.942	(-0.92; 0.85)
Graduation country	MENA countries						
	South Asian countries	**-0.56**	**0.047**	**(-1.11; -0.01)**	**-0.66**	**0.028**	**(-1.25; -0.07)**
	East Asian and European countries	0.18	0.768	(-1.02; 1.38)	-0.21	0.72	(-1.38; 0.95)
Pharmacy location	Abu Dhabi						
	Dubai	**1.02**	**0.004**	**(0.34; 1.7)**	**0.90**	**0.009**	**(0.23; 1.56)**
	Sharjah	0.45	0.24	(-0.3; 1.19)	**0.87**	**0.021**	**(0.13; 1.61)**
	Other emirates (UAQ, RAK, Ajman, and Fujairah)	0.68	0.088	(-0.1; 1.47)	**1.11**	**0.008**	**(0.29; 1.93)**
Pharmacy type	Independent pharmacy						
	Chain pharmacy	**0.91**	**0.002**	**(0.32; 1.49)**	**0.88**	**0.004**	**(0.29; 1.46)**
Current employment	Pharmacist						
	Pharmacist technician / Trainee	-0.77	0.109	(-1.71; 0.17)	-0.36	0.446	(-1.28; 0.56)
Work experience		**0.08**	**0.003**	**(0.03; 0.13)**	**0.13**	**0.001**	**(0.05; 0.2)**
Number of pharmacists in the pharmacy	1 pharmacist						
	2 pharmacists	0.83	0.185	(-0.4; 2.06)	0.89	0.144	(-0.31; 2.09)
	≥ 3 pharmacists	0.84	0.171	(-0.36; 2.04)	0.81	0.176	(-0.36; 1.98)
Education on CAM included in undergraduate pharmacy curriculum	No						
	Yes	**0.83**	**0.008**	**(0.21; 1.44)**	**0.68**	**0.026**	**(0.08; 1.29)**
Received post-graduate education/training on CAM products	No						
	Yes	0.30	0.286	(-0.25; 0.85)			
Perceived usefulness of education/training on CAM products	Not Useful	-0.56	0.611	(-2.72; 1.6)	-0.94	0.371	(-3; 1.12)
	Neutral						
	Very Useful	0.62	0.147	(-0.22; 1.46)	0.37	0.379	(-0.46; 1.2)
	No answer	-0.03	0.94	(-0.82; 0.76)	0.04	0.927	(-0.74; 0.82)
Willingness to attend trainings/ workshops conducted on CAM products	No						
	Yes	0.56	0.113	(-0.13; 1.24)	0.27	0.447	(-0.43; 0.97)
CAM primary source of knowledge	Social Media / Family & friends						
	Education / Training / Work Experience	**0.97**	**0.005**	**(0.3; 1.64)**	**0.79**	**0.021**	**(0.12; 1.45)**

Values in bold are statistically significant

## Discussion

This study provided new insights into CPs’ knowledge, attitudes, and practices regarding CAM use. The findings have implications for patient safety, outcomes, policy development, and continuous professional development.

Overall, the surveyed CPs had a good knowledge with regards to the functions of the CAM products, however, there was limited knowledge about CAM’s safe use, as evidenced by their incorrect responses concerning potential side effects and drug interactions. More than half of the pharmacists provided incorrect responses when questioned about the safe usage of ginseng in individuals with hypertension. This lack of awareness is concerning, especially in light of the high prevalence of hypertension in the UAE, inadequate awareness about the condition, and its widespread suboptimal control [29]. Moreover, over two-thirds of the pharmacists were unaware of the increased risk of bleeding associated with combining gingko with Coumadin, a widely used medication for treating and preventing several cardiovascular conditions [30]. These findings align with similar global and local studies, highlighting a consistent pattern of insufficient knowledge among pharmacists regarding CAM’s side effects [26, 31–33]. Pharmacists’ knowledge gaps regarding CAM’s side effects can result in suboptimal advice and jeopardize patient health and safety, as highlighted in a study by Hijazi et al. [32].

In line with the relatively good knowledge of CPs, the results of this study showed that pharmacists in the UAE generally hold a positive attitude towards CAM utilization, which is in agreement with previous studies conducted in various regions worldwide [34–36]. About 3-in-4 pharmacists acknowledged the widespread use of CAM products in the country and recognized their potential to impact general health outcomes positively. These outcomes highlight the importance of integrating evidence-based CAM practices into pharmacy services in the country to meet the evolving healthcare needs of the population. While CPs reported a positive attitude towards CAM, they also exhibited an incongruous perspective concerning their professional responsibility in providing knowledge about CAM to the public. Three-quarters of the pharmacists surveyed stated that providing information about herbal medicine falls outside their professional responsibility. This viewpoint has significant implications for patient safety. Such attitudes could result in inadequate guidance on the safe use of CAM, adverse effects, and potential associated risks. In a study conducted by Oi Lam Ung et al., interviewed pharmacists expressed the belief that providing services for dietary supplements fell outside their scope of practice. Consequently, they did not integrate awareness of their uses into their daily practice or ensure the safety of the stocked supplements, potentially compromising the safety of their patients [37].

The practices related to CAM reported in this study highlight the significant involvement of CPs in CAM, given the high proportions of CPs who sold CAM, received inquiries about CAM and advised consumers on how the CAM products ought to be used. However, sizable proportions did not ask about the medical history of the patients, the drugs that could potentially interact with the CAM product being used nor the possible side effects and toxicity levels. Approximately one-third of the surveyed pharmacists disclosed that they occasionally or infrequently inform consumers about the potential adverse effects, risks, and interactions associated with using CAM products. The limited frequency of comprehensive counselling provided by CPs is likely due to their insufficient knowledge in this area, posing a significant concern. This situation heightens patient safety concerns, particularly given the widespread utilization of CAM and the diverse population and range of products used in the UAE [25]. These findings align with previous studies that have shown that CPs in the UAE do not typically offer counselling about medication side effects unless asked, and even then, only a small number provide thorough counselling [38, 39]. A study by Mobark et al. reported that the overall counselling of the participating pharmacists was inadequate and that none of them provided spontaneous counselling on medication side effects; such practices may compromise the quality of medicine, adherence to treatment, and patient satisfaction [38].

Pharmacists’ limited knowledge and skills regarding evidence-based CAM may limit their capacity to provide accurate and reliable information about its potential risks and inappropriate use. This could make them hesitant to discuss CAM options with patients, which limits the opportunity for meaningful discussions and shared decision-making. A study conducted by Moin and colleagues found that a pharmacist-led shared decision-making intervention improved patients’ understanding of their treatment options and led them to choose the options that aligned best with their values and preferences, resulting in improved adherence to treatment and improved treatment outcomes [40]. Moreover, the lack of comprehensive counselling and limited opportunity for shared-decision making may prompt patients to seek advice from family and friends, influenced by the collectivistic nature of the Arab community. A study conducted among UAE citizens in Abu Dhabi revealed that the main influences on their CAM purchasing patterns were the advice and recommendation of family and friends [25]. This reliance on the advice of non-professionals poses a risk of CAM misuse.

As the healthcare landscape evolves, pharmacists must stay up-to-date with their patients’ growing use of CAM products and have appropriate communication skills to effectively address any concerns related to the selection and use of these products. In a study involving pharmacy students during their advanced pharmacy practice experience, they were taught to approach the topic of CAM use sensitively by providing examples of commonly used products in Hispanic culture and ensuring that their patients understood the reasoning behind the questions asked. This helped the students develop new skills in providing culturally competent care, highlighting the importance of integrating CAM education into pharmacy school curricula, including professional placement settings [41].

The results of this study showed that CPs in chain pharmacies exhibited higher knowledge scores than those employed in independent pharmacies. This disparity could be attributed to several factors. Firstly, chain pharmacies often provide ongoing training programs for their pharmacists, enabling them to stay updated with the latest advancements in the field. Additionally, chain pharmacies are more likely to implement patient-oriented initiatives and services, further enhancing pharmacists’ knowledge [38]. Another contributing factor could be the competitive pricing strategies chain pharmacies employ for CAM products, in contrast to independent pharmacies [42]. As a result, individuals are more inclined to visit chain pharmacies for their CAM needs, leading to greater exposure for pharmacists employed in these settings to diverse populations. This exposure affords them invaluable opportunities to accumulate extensive experience and knowledge about CAM.

The results of this study show that pharmacists who perceived themselves as having more knowledge had higher knowledge scores. This suggests that pharmacists can recognize their knowledge gaps. However, despite this self-awareness, almost 3-in-4 of surveyed pharmacists disagreed with including CAM in pharmacy school curriculums. This disagreement may cause missed opportunities for pharmacists to learn about CAM and provide better patient care. In a study by Simpson et al., CAM was increasingly incorporated into undergraduate pharmacy education to provide pharmacy students with the knowledge and skills required to best advise consumers on the safe and effective use of CAM. Feedback from students indicates that this approach enhanced their understanding of the potential adverse effects associated with CAM products and enabled them to decide on the suitability of certain CAM products for different disease states and with different drugs [43]. Similarly, in this study, participants’ knowledge scores were higher if they were educated on CAM during their undergraduate studies. These findings emphasize the necessity of integrating CAM into a structured curriculum, particularly within undergraduate studies of universities in the UAE, to ensure that pharmacists receive adequate knowledge of the widely used CAM therapies. Currently, in the UAE, there exists a specific framework for pharmacy graduate (UAE Professional Pharmacy Graduates Competency Framework) that addresses the competency standards for professional pharmacy programs in the UAE [44]. This framework was developed through a participatory approach involving the Commission for Academic Accreditation, representatives of colleges of pharmacy in the country, stakeholders from professional practice, and government regulators. Although this framework does not address the role of pharmacists in CAM specifically, it lays out many competencies related to public health and patient safety which relate significantly to CAM. As a result, pharmacy schools have responded to this framework by introducing topics and workshops on CAM over the past decade. Initially, CAM subjects were only introduced to a limited extent. However, more recently, pharmacy schools in the UAE have aimed at strategically integrating CAM education into their curricula. Some schools now offer courses or topics on herbal medicine, acupuncture, and other CAM modalities alongside conventional pharmacy subjects. This training helps students learn how to evaluate evidence critically, understand potential interactions and ensure patient safety when dispensing CAM. Nonetheless, it is essential to place more emphasis on practical skills and experiential learning in addition to theoretical knowledge. A recent review of pharmaceutical care education at pharmacy colleges in the MENA region recommended that recommendations improvements in the pharmacy curricula to support pharmacy students’ preparation to become more competent patient care practitioners [45]. The aforementioned recommendation regarding the integration of CAM within the academic pharmacy curricula in the UAE targets pharmacists who study in the country. However, the fact that over 60% of participants did not receive their undergraduate degree from the UAE calls for other remedial measures. For instance, CAM education could be offered as part of required continuing development activities or as adjunct courses to license renewals.

Surprisingly, in this study, there was no difference in knowledge between participants with and without a pharm D degree. Although it was expected that those with Pharm D would have better knowledge given their more rigorous training in clinical patient care [46]. This finding highlights the need to integrate knowledge and good clinical practice within the Pharm D as well as the pharmacy curricula and professional development activities in the UAE.

The study’s findings indicate a significant relationship between knowledge and attitudes among pharmacists (*r* = 0.108, *p* = 0.036). However, no significant association was observed between knowledge, attitudes, and practices, indicating that pharmacists’ practices may not be primarily influenced by their knowledge and attitudes (micro-level factors). Instead, it is possible that external factors (meso and macro-level factors) such as policies, rules, and frameworks governing the scope of pharmacy practice in the country, workload, managers support, and funds play a more prominent role in shaping pharmacists’ practices. Further research and exploration of these policies and frameworks could provide a deeper understanding of the factors influencing pharmacists’ practices [47–49]. .

This study has several strengths. Firstly, it was a national cross-sectional survey that covered all seven emirates in the UAE, and it used a stratified random sampling approach, which ensured representation across different regions and health authorities. This enhanced the validity and generalizability of the findings and minimized the potential for bias. Moreover, the study examined various aspects of community pharmacists’ knowledge, attitudes, and practices regarding CAM use, providing a broad understanding of the subject. However, the study has some limitations that must be considered. It relied on self-reported data from community pharmacists, which may have been influenced by social desirability bias, leading to a potential overestimation of their knowledge, attitudes, and practices. Moreover, the study could not completely unearth the causes of the pharmacists’ insufficient knowledge in specific areas, some negative attitudes, and suboptimal practices toward CAM use.

## Conclusion

The findings of this national investigation showed that CPs in the UAE hold a generally positive attitude towards CAM utilization. Such a positive attitude is coupled with good knowledge about the health indications of several CAM products. However, there was limited knowledge among surveyed CPs about CAM’s safe use and contraindications. The pharmacists’ knowledge gap in this area was also reflected in their practices, as they seldom counsel their patients on CAM’s side effects. A main opportunity identified in this study is the fact that pharmacists are mostly aware of their knowledge gaps. In this context, the findings of this study showed that pharmacists working in independent pharmacies, those with fewer years of experience and those who did not receive CAM education during their academic degree have lower knowledge. Together, the results of the study provide critical evidence for the development of targeted interventions to improve the knowledge as well as the practices of pharmacists with regards to CAM. For instance, plausible interventions include integrating CAM education within the curricula of pharmacy in the country as well as within professional development activities and possibly license renewal. Such activities could address CAM safety and contraindications, patients’ counselling techniques, CAM indications for specific health conditions, and evidence-based CAM information retrieval. In addition to education interventions, a system-based approach is needed to support pharmacists in promoting the safe and effective use of CAM and in reporting side effects. Future research is needed to examine the effectiveness of such interventions in supporting CPs’ role in ensuring a safe and effective use of CAM and in improving patients’ outcomes in terms of patient understanding of CAM use, adherence to recommendations and health indicators.

## Data Availability

The raw data can be made available upon reasonable request from the corresponding author.

## Abbreviations

ACCP: American College of Clinical Pharmacy
ASHP: American Society of Health-System Pharmacists
CAM: Complementary Alternative Medicine
CI: Confidence intervals
CPs: Community pharmacists
MENA: Middle East and North Africa
OTC: Over-the-counter
POM: Prescription Only Medicines
SD: Standard Deviation
UAE: United Arab Emirates
